# Ventilation

**Published:** 1872-11

**Authors:** 


### HALL'S

### JOURNAL OF HEALTH.

VOL. XIX. — NOVEMBER, 1872. — No. XI.

### VENTILATION.

MRS. ARABELLA WILLSON, of Canandaigua, New York, deserves great credit for the very original manner in which she has drawn public attention to the subject of ventilation, especially in churches; and we join the *Christian Weekly* in the hope that her keen wit and royal good sense will greatly aid in effecting a much needed reform. The appeal is from the original manuscript, word for word: —

### A APELE FOR ARE TO THE SEXTANT.

BY A GASPER.

O Sextant of the meetinouse, which sweeps
And dusts, or is supposed to ! and makes fiers,
And lites the gas, and sumtimes leaves a screw loose,
In which case it smels orful — wus than lampile :
And wrings the Bel and toles it, and sweeps paths ;
And for these servases gits $100 per annum ;
Wich them that thinks deer let em try it ;
Gittin up before starlite in all wethers, and
Kindlin fiers when the wether is as cold
As zero, and like as not green wood for kindlins,
(I wouldnt be hierd to do it for no some ;)
But O Sextant there are one kermodity

Wuth more than gold, wich dont cost nothin;
Wuth more than anything except the Sole of Man!
I mean pewer Are, Sextant, I mean pewer Are!
O it is plenty out o dores, so plenty it doant no
What on airth to dew with itself, but flize about
Scaterin leaves and bloin off men's hats;
In short its jest as free as Are out dores;
But O Sextant! in our church its scarce as piety,
Scarce as bankbils when ajunts beg for mishuns,
Wich sum say is purty often; taint nothing to me,
What i give aint nothin to nobody; but O Sextant!
You shet 500 men women and childern,
Speshily the latter, up in a tite place, —
Sum has bad breths, none of em aint too sweet,
Sum is fevery, sum is scroflus, sum has bad teath,
And sum haint none, and sum aint over clean;
But every one of em brethes in and out and out and in
Say 50 times a minnet, or 1 milion and a half breths an hour;
Now how long will a cherch full of are last at that rate?
I ask you; say 15 minnets, and then whats to be did?
Why then they must brethe it all over agin,
And then agin and so on, til each has took it down
At least 10 times and let it up agin, and whats more
The same individible doant hev the privilege
Of brethin his own are and no ones else,
Each one must take wotever comes to him.
O Sextant! doant you no our lungs is bellusses
To blo the fier of life and keep it from
Goin out; And how can bellusses blo without wind?
And aint wind are? I put it to your conshens.
Are is the same to us as milk to babies,
Or water is to fish, or pendlums to clox,
Or roots and airbs unto an Injun Doctor,
Or little pills unto an omepath,
Or Boze to gurls. Are is for us to brethe.
What signifize who preeches ef I cant brethe?
Whats Pol? Whats Pollus to sinners who are ded
Ded for want of breth? why Sextant when we dye
Its only coz we cant brethe no more — thats all.
And now O Sextant! let me beg of you
To let a leetle are into our cherch;
(Pewer are is serting proper for the pews,)
And dew it week days and on Sundys tew —
It aint much trubble — only make a hoal
And then the are will come in of itself.
(It loves to come in where it can git warm.)
And o how it will rouze the peeple up
And sperrit up the preecher, and stop garps

And yorns and fijjits as effectooal
As winds on the dry Boans the Profit tels
Of. MRS. ARABELLA WILLSON.

The *Christian Weekly* gives the Sextant's reply, thus:—

My dear Miss Willson, if you only knew
How hard my fate is, you would surely have
A little mercy on me. I know how good it is
To have pewer Are to breathe, but how on airth
Am I to get it in? The pewer Are won't come in
Unless there is some hole or opening for it.
And I can't make it, no how. There are the dore
And winders, it is true. But if I open my dore,
Or leave one open, just ajar, and do it on the sly,
To let a little pewer Are come in,
It creeps around the people's feet, and then
They call it a cold wind, and not a pewer Are.
And if I open down the winder tops
There always is by chance some fidgety old lady
Or some man that has the rumatiz, or else
Some venerable baldhead that it comes down upon;
Then there comes a breeze which blows me up,
And is not like at all a breeze of pewer Are.
They sometimes put up in the ceilin a winder
What people call a "ventilator," to let the poor are out.
The simple souls don't seem to know at all
That poor are can't go out, unless the other can come in
To take its place; and that it can't come in
Without some opening. I can't make it nohow.
So what shall a poor sextant do with people
That say they must have pewer Are
But wont allow him nary chance
To get it in? Let 'em only let me know
How they will have it—'round their feet
Or on their heads and shoulders,
And then we'll all agree on one or tother.
Or else if they'll call a meeting
And vote some better plan, and pay the cost of it,
Not for the poor are to get out—that's easy—
But for letting pewer Are in. Let 'em do that,
And stop worritting the poor sextant,
Because he can't make merchantable bricks
Without no stror.

That pure air is more essential to health and life than food or drink is demonstrated by the fact that we can live for days without drink, and weeks without food, but only a few minutes without air. A most dreadful narration, illustrating this point, is given in the words of a sufferer in the Black Hole of Calcutta, in "Sleep, or Hygiene of the Night," one of the publications of Hurd and Houghton, 13 Astor Place, New York. And as we are in our houses four fifths of our time in winter, it is of incalculable interest to have a pure air to breathe during that time, and we do well to do all we can to find out how to get the purest possible. Theories are as numerous as the patent stoves, heaters, and furnaces, devised for that purpose; each latest patent may have an advantage beyond any of its predecessors; but until greater perfection is reached, we may as well make up our minds to the simple fact that the very best and most healthy system of ventilation may be seen in a little old stone house, overlooking the banks of the beautiful Hudson, at Newburgh, in which General Washington once, undoubtedly, ate and slept for many a day and night.

The fire-place occupies a greater part of one side of the building, and the mode of procedure was to kindle an immense wood fire, and keep the front door open. This system of ventilating and warming family rooms has never been excelled, and may not be in our day.

The next best plan, perhaps, is, as a general rule, in cities and towns, to sleep in the upper rooms of the building, with such an arrangement as will allow the air of the lower part of the house to be shut off by means of a single door at the foot of the stairs which lead to the upper floor; this will prevent the heat of the lower part from being wasted in the sleeping-rooms, and also afford an excellent opportunity of airing the chambers for several hours in the middle of every day during which it may not rain; with another advantage, that the various odors and gases and other impurities, from the cellar and kitchen and dining-room, cannot befoul the chamber air.

Then, whatever may be the method of warming the house, whether by grates, or heaters, or furnaces, or fire-places, let every inner door be kept wide open, and then keep the fires burning with sufficient freedom to secure healthful warmth; the door leading to the cellar being always kept closed, the cellar ceiling being well plastered, and the space between it and the floor to be well filled with dry ashes or other material, to keep the emanations and dampness of the cellar from rising up into the family room, through the crevices in the floor.

In this way, there will be quite enough out-door air coming in at the window and door crevices to keep up a good ventilation, while it is warmed in its passage through the halls, and thus enters the family room without a draught.

There is no advantage to any one in sleeping in a room colder than fifty degrees Fahrenheit; colder than that is positively dangerous to all old and sickly persons, and young children; hence, in very cold weather, the door leading to the upper story, where the chambers are, should be opened before retiring, and remain open until morning; this will temper the air of the chambers, and modify any dampness in them.

A good sized grate will consume about four tons of coal, with ordinary usage, during the winter, and every family able to afford it should have a wide open fire in the family room, kept cheerfully burning, especially before breakfast and after supper; it has a cheering, enlivening effect on the whole household, and will well pay the cost in that direction.

The best device known for keeping up a good fire in a large fire-place, with a wide throat leading up into the chimney, giving a broad surface of burning coals on the hearth, on a level with the floor, is Dixon's patent, of Philadelphia.

As to the proper method of

HEATING CHURCHES, there are several suggestions of considerable practical importance.

First. The proper measure of heat for cold weather is about sixty-five degrees Fahrenheit, about four feet above the floor. At the close of the services of the day, all the windows and doors should be opened, and kept open for two or three hours, so that the draughts of air passing through the building should carry with them, out of doors, the immense amount of human emanations and other impurities which are held in the air of any public building, after its occupancy. If this is not done, these odors solidify in part, and dry on the walls and glass and wood-work, to be reconverted into fumes when the fires are next kindled, and to be rebreathed. It is a great mistake to suppose that it is sufficient if the house is ventilated, without heat, during the week, or just before warming up again for the next meeting, for the reason just named.

Second. In the common churches in the country in winter time, good fires ought to be lighted on Saturday afternoon, and kept up until service next day, in order to get rid of the dampness and closeness observed in all unoccupied buildings. In city churches which are larger, fires are kindled in very cold weather on Fridays, and are kept burning until the Sabbath service.

With attention to these matters, and open doors and windows between the Sabbath services, any other ventilation can be dispensed with, if one thing is done: reduce the service to one hour. This can be easily done if the useless introductions in the sermon are dispensed with, as also the unseemly "notices" at the conclusion of the services, including valuable time, worse than lost in organ screeching between the verses, for the glorification of the organist. Only think of keeping a thousand or two people listening against their will, to one, two, three, and even four lines of amateur freaks of fingering. Everybody knows that the organist is simply trying to show himself off, and that too, when perhaps not one in ten of the congregation ever heard of his name, or ever had interest enough to ask the sexton what it was.

It is not an uncommon thing to note that between these three useless things, introductions, notices, and organ thrumming, fifteen minutes of every public service are lost; worse than lost, for the people are breathing bad air that much longer, unnecessarily.

There is another method of antagonizing the ill effects of imperfect ventilation in churches on Sundays, never thought of before, in all probability, since the Creation.

Most readers will remember its having happened before now, that having eaten a very hearty dinner of a summer's day, or in a warm room at winter time, there was an irrepressible desire to get into the open air, and a sense of most grateful relief on being able to do so. The reason is that the stomach was so full it pressed up against the lungs, crowded them, prevented their getting the needed amount of air, and there was a feeling of more or less imminent suffocation. And if, with such a full stomach, we go to the impure air of a crowded church, it is no wonder we feel dull and stupid, with about as much feeling of devotion and interest in the sermon as one of the stone pillars. If, however, one goes to meeting, having taken a light meal, say a cup of tea and a sandwich, as much of them as he wants, there will be an amelioration of the consequences of a want of ventilation, which will be a surprise to any one intelligent enough to make the experiment; and there will be no disposition to growl at the uninterestingness of the sermon, or the carelessness of the "Sextant."

